# Metal-based (lignin/silica) hybrids as green activators for conductive EPDM composites

**DOI:** 10.1038/s41598-025-34534-x

**Published:** 2026-01-27

**Authors:** Khlood S. Abdel Zaher, Doaa S. Mahmoud, Salwa H. El-Sabbagh, A. A. Ward, Galal A. M. Nawwar

**Affiliations:** 1 Green Chemistry Department, National Research Centre, 33 El-Bohouth Street, Dokki, Giza, 12622 Egypt; 2 Polymers and Pigments Department, National Research Centre, 33 El-Bohouth Street, Dokki, Giza, 12622 Egypt; 3 Microwave Physics and Dielectrics Department, National Research Centre, Cairo 33 El-Bohouth Street, Dokki, Giza, 12622 Egypt

**Keywords:** EPDM elastomer, Rice straw, Green activator, Electrical conductivity, Chemistry, Engineering, Environmental sciences, Materials science

## Abstract

Conductive rubber composites are usually formulated from natural or synthetic rubber and a variety of petroleum-based additives. Environmental and sustainability concerns have increased interest and resulted in the search for bio-based alternatives to these additives. Rice straw is an abundant agricultural waste whose burning contributes to environmental pollution. It can be transformed into valuable resources for green composites in a sustainable manner. This research presents a sustainable approach to upcycle this biomass by developing metal–organic hybrid materials, Fe(lignin/silica/fatty acid) Fe(LSF) and Ni(lignin/silica/fatty acid) Ni(LSF) hybrid materials from rice straw black liquor for use as green activators in ethylene propylene diene monomer (EPDM) rubber composites. These hybrids were thoroughly characterized, the X-ray fluorescence spectroscopy (XRF) and X-ray diffraction (XRD) investigations confirmed the crystallinity of the Fe(LSF) hybrid and the amorphous nature of the Ni(LSF) hybrid, as well as determining the elemental composition. In addition, the morphology at the nanoscale and uniform distribution of the elements in both hybrids were confirmed by transmission electron microscopy (TEM), scanning electron microscopy (SEM), and EDX mapping analysis. The hybrids were then added to EPDM formulations to replace the traditional activator system of stearic acid and zinc oxide (ZnO). The performance of the composites was then evaluated, revealing that the green activator systems impart interesting properties. The vulcanizates achieved tensile strengths up to 5.57 MPa, showed improved resistance to thermo-oxidative aging, and demonstrated enhanced electrical conductivity. These results underscore the potential of these rice-straw-derived hybrids as sustainable, high-performance components for electrically functional EPDM applications.

## Introduction

Greater concern about global warming and climate change has prompted efforts to improve energy efficiency and environmental sustainability. Thus, researchers have targeted agricultural wastes that contain natural fibers due to their renewable and biodegradable properties^1^. Agricultural waste management in countries primarily dependent on crops is becoming a major topic of discussion because of its adverse environmental effects, the positive aspect of recovering resources and the sustainability benefits that come with it. In particular, growing awareness focuses on mitigating air pollution from open-field burning and improving waste utilization through circular economy approaches that convert residues into higher-value materials like biochar, bio-activators, or composites^2^. In Egypt, the practice of open burning of rice straw, a low-value agricultural residue is an important environmental issue that contributes significantly to air pollution, soil degradation and threats to health^3^.

In response, current research is focused on the sustainable valorization of rice straw agricultural waste stream to extract valuable components such as lignin and silica to use in potential applications in the rubber, paper, and agricultural industries^4–7^. Lignin-driven advanced materials provide a sustainable and economically viable pathway for lignocellulosic biomass utilization, integrating environmental and economic assessments to guide responsible material development^8^. This work aims to valorize black liquor, a by-product of solar pulping of rice straw, by converting its (lignin, silica, and fatty acids) components into advanced hybrid materials with potential applications in rubber composites. Alkaline precipitation for black liquor by promoting metal–organic interactions, utilizing metal salts such as ZnCl_2_, AlCl_3_, and CuSO_4_ offer a simple and cost-effective route for synthesizing hybrid metal–organic complexes with broad industrial applications^9,10^. To satisfy consumer needs, the rubber industry is continually looking for materials that are affordable, long-lasting, and environmentally friendly^11^. This is consistent with the widely recognized concept of sustainable development, which encourages conscientious material and economic advancement while protecting the environment and human health by using natural resources wisely for the benefit of future generations. The composition of rubber products has a significant impact on their performance^12,13^.

Recently, rubber-based composites have attracted great attention due to their unique combination of flexibility, resilience, and multi-functionality for use in automotive, electrical, and electronic applications. Functional additives are important in terms of enhancing the mechanical, thermal, and electrical performance of rubber matrices, such as EPDM, NBR, and silicone rubber^14^. Conductive green rubber composites are one interesting class of materials in this space that provide elasticity along with electrical conductivity, enabling their application in antistatic components, electromagnetic interference shielding, flexible sensors, and wearable electronics. Zinc oxide remains a commonly used conventional activator in rubber vulcanization; however, growing environmental concerns have driven efforts to identify more sustainable substitutes. Currently, researchers aiming for eco-friendly, cost-effective, and scalable solutions for sustainable development continue to confront one of the major ongoing challenges in the field^15,16^. It is preferable to minimize ZnO concentration in order to save production costs and minimize contamination hazards. To reduce the amount of zinc in elastomer composites, several strategies have been investigated.

Wang et al. produced core–shell ZnO nanoparticles via wet precipitation utilizing a variety of cores, resulting in reduced ZnO content with greater vulcanization efficiency and mechanical reinforcement in NR/SBR/BR. Due to improved dispersion and interfacial contact^17^. Sugarcane-derived activators were investigated by Zanchet et al. as potential substitutes for stearic acid and ZnO in natural rubber. With encouraging results, this substitution changed the vulcanization process, impacting the structure, cross-link density, mechanical characteristics, and thermal stability^12^.

Wu et al. studied replacing zinc oxide in commercial styrene-butadiene rubber (SBR) formulations with amine-passivated carbon dots (CDs). Their results showed that using CDs instead of ZnO greatly reduces the activation energy of sulfur cross-linking processes, which promotes the development of sulfur-based networks in SBR composites^18^.

In nitrile rubber, Silva et al. investigated substituting commercial magnesium oxide and a green magnesium oxide derived from renewable resources for zinc oxide. According to the findings, employing green magnesium oxide as an activator is a viable and efficient choice^19^. A crucial synthetic elastomer that was first used in the 1960s, ethylene propylene diene monomer (EPDM) rubber is a synthetic elastomer known for its excellent electrical insulation, sealing capacity, and weather resistance. While EPDM rubber has good thermal stability, this elastomer is very flammable and produces toxic smoke when ignited. For this reason, EPDM rubber is being studied to improve functional properties for these applications, such as flame retardancy and electrical conductivity^20^.

Previous studies have highlighted the potential of complexes derived from rice straw black liquor to be used as multifunctional rubber additives. Lignin/silica and calcium lignate/calcium silicate were examined by Abdel Zaher et al. as natural antioxidants in vulcanizates of styrene-butadiene rubber (SBR). They discovered that 8 phr of these additives exceeded traditional antioxidants like TMQ and IPPD and produced the best mechanical characteristics in SBR vulcanizates^9^.

Othman et al. reported that incorporating Cu(LSF) into NBR acted as an antioxidant and a conductive additive, increasing ionic pathways and improving electrical conductivity, hardness, and fluid resistance in vulcanized rubber^21^. Abdel Zaher et al. studied Zn(LSF) from rice straw black liquor as an activator and antioxidant in NR composites. Zn(LSF) effectively replaced ZnO/Zn-stearate and TMQ, serving as both a vulcanization accelerator and a reinforcing stabilizing additive^22^. Mahmoud et al. studied the preparation of flexible, conductive composites by blending an Al(LSF) hybrid filler with natural rubber. The electrical conductivity of the resultant rubber composites was greatly enhanced by this combination^23^. In this work, renewable natural compounds from black liquor mainly lignin, silica, and fatty acids were combined with transition metals. Nickel and iron were selected for incorporation into rubber composites because they form stable hybrids with lignin and silica, thereby acting as effective activator, while enhancing the composites’ thermal stability and electrical conductivity.

With this strategy, the research aims to continue searching for the efficiency of the metal/lignin hybrids as green activator in elastomer formulations, with the goal of reducing or eliminating the use of conventional, environmentally harmful activators. A systematic investigation was conducted to assess how hybrids affect the mechanical, conductive, curing, and thermo-oxidative degradation properties of EPDM composites, alongside a comparative study with the conventional activator system of ZnO and stearic acid.

## Experimental

### Materials

The black liquor used in this study was obtained from the alkaline solar pulping of rice straw, collected from El-sharkia government, Egypt. The rice had an average length of approximate length of 1.0 m.

The pulping experiment of rice straw was carried out in closed polyethylene bags following the procedure described in our patent^24^. Sodium hydroxide (NaOH) was used as the pulping chemical at a concentration of 20% (w/w) based on the dry weight of the straw, with a liquor-to-fiber ratio of 10:1. The sealed bags were exposed to direct sunlight as a natural heating source until the fibers were fully cooked. After the solar treatment period, the resulting black liquor was collected for further use, while the pulped fibers were thoroughly washed with water until neutral pH was achieved and then air-dried at room temperature.

Analytical-grade ferric chloride anhydrous (purity: 98.5%), nickel (II) sulfate hexahydrate (purity: 98.5%), and sodium hydroxide were as received, supplied from BDH, Merck, and Sigma-Aldrich. All other chemicals and solvents employed were of laboratory reagent grade.

In this formulation, the EPDM rubber (Vistalon 650S, ESSO Chemie, Germany) was used as the base polymer. It contained 55% ethylene and 9% ethylidene norbornene, had a density of 0.86 g/cm^3^ and exhibited Mooney viscosity ML (1+4) at 127 °C ranging from 48 to 52. In order to increase processability and performance, naphthenic processing oil was utilized as a plasticizer. CBS (purity 98%) obtained from RheinChemie, Germany, was applied as a vulcanization accelerator. The activators stearic acid (98.5% purity) and zinc oxide (ZnO) (99.9% purity) exhibited specific gravities ranging from 0.9-0.97 and 5.55-5.61, respectively. Also, it supplied from Aldrich (Germany). Elemental sulfur was used as the vulcanizing agent while TMQ, was used as an antioxidant, also supplied by Aldrich (Germany).

### Fe(LSF) and Ni(LSF) hybrids preparation

15 g of anhydrous ferric chloride were added to 1L of rice straw pulping black liquor (pH 12) under condition stirring. The pH decrease to around 4, and the liquor was left to stand overnight, the resulting precipitate was the filtered, washed with tap water and oven dried it at 105 °C, yielding about 37 g of dark brown powder.

Similarly, 40 g of nickel (II) sulfate hexahydrate, was added to another 1L of rice straw pulping black liquor (pH ≈ 12) under stirring. After the resulting liquor reached (pH 7) and stand overnight, 31 g of dark brown powder were obtained following by filtration, washing, and drying at 105 °C^23^.

### EPDM compounding

The environmentally friendly activators Fe(LSF) and Ni(LSF) hybrids were studied as potential activators for EPDM vulcanization, in view of conventional systems of zinc oxide and stearic acid. The rubber formulations were based on ASTM D3182-07(2012) (Table 1). Initially, the EPDM compounds were mixed on a two-roll mill. Stearic acid and zinc oxide were added to the EPDM matrix and blended thoroughly as conventional activators. Subsequently, the accelerator (CBS), antioxidant (TMQ), and the elemental sulfur were incorporated. The entire mixing process took around 20 min at ambient conditions. Afterward, a desired amounts of Fe(LSF) and Ni(LSF) hybrid activators were introduced into the EPDM compounds. This was followed by the addition of CBS, the TMQ, and elemental sulfur. The resultant compounds were then molded using a hot-press at 152 ± 1 °C for their optimum vulcanizing times, as determined by MDR one moving die rheometer.

**Table 1:** Formula for EPDM composites.

Samples Codes	E0	EF1	EF2	EF3	EF4	EN1	EN2	EN3	EN4
EBDM	100	100	100	100	100	100	100	100	100
ZnO	5	2.5	–	5	–	2.5	–	5	–
St. acid	2	1	2	–	–	1	2	–	–
Fe(LSF)	–	3.5	5	2	7	–	–	–	–
Ni(LSF	–	–	–	–	–	3.5	5	2	7

Base recipe (in phr): Ethylene propylene diene monomer (EPDM) 100; CBS 0.8; Naphthenic processing oil 2; S 2.5; TMQ 1.

### Characterization

The XRF analysis elemental analysis by wavelength dispersive X-ray fluorescence spectrometry using Axios advanced, Sequential WD_XRF spectrometer, PANalytical 2005.

XRD data were measured by the modern diffractometer Bruker d8 advance, Germany, using copper source Kα radiation (λ = 1.5406 Å) at 40 mA, 40 kV, in the 2θ range 5°-80°, step size 0.05° using automatic divergence slit and scan rate of 0.6°/sec.

The crystallinity Index (*CrI* %) index was calculated by the XRD peak-deconvolution integration (area) method, according to Eq. (1). The diffractograms were deconvoluted into crystalline and amorphous components using Origin Pro, and peak areas were obtained after background subtraction and curve fitting (Gaussian functions).

1 $$CrI\left(\mathrm{\%}\right)=\left\{{A}_{crystalline}/\left({A}_{crystalline}+{A}_{amorphous}\right)\right\} \times 100$$

where ${A}_{crystalline}$ is the summed area of fitted crystalline peaks and ${A}_{amorphous}$ is the area the summed area of the amorphous halo in the diffractogram^25^.

TEM for Fe(LSF) and Ni(LSF) hybrids were investigated by A JEOL JEM 2100 transmission electron microscope with a micro analyzer electron probe (JEOL Co. Ltd., Japan). After three hours of drying at 70 °C in an oven, the hybrid powders were suspended in 0.02 weight percent ethanol and subjected to a 15-min bath sonication. Prior to imaging, the suspension was dropped several times onto carbon-coated copper grids (300 mesh), and any excess was blotted with filter paper before being allowed to air and then vacuum-dry. The particle dimensions (length and width) were measured from TEM images using ImageJ software. At least 30 particles were analyzed to determine the average length, width, and aspect ratio (AR = length/width)^26^.

FTIR was evaluated on a JASCO FTIR-6000 E (Japan) with wavenumber range from 400 to 4000 cm^-1^ at a scanning resolution of 4 cm^**-**1^. EPDM composites, on the other hand, were analyzed with a Model ATR PRO450-S, single reflection measuring attachment. The morphologies of the hybrids and rubber specimen were characterized by JEOL (JXA-840A) electron probe microanalyzer, Japan. Equipped with energy-dispersive X-ray spectroscopy (EDS). The cross section of the rubber specimen was coated with a small layer of gold after it was fractured in liquid nitrogen.

In accordance with the ASTM D 2084-07, 2007a standard, each sample was weighed and the curing characteristics were assessed using a Monsanto oscillating disc rheometer-100 at 152°C for duration of 40 min. According to ASTM D412-06a (2013) standard, the mechanical properties of the Fe(LSF) and Ni(LSF) hybrid /EPDM composites were tested using a dumbbell shaped specimen on a Zwick testing equipment machine, with a tensile rate of 500 mm/min. Thermo-oxidative aging of EPDM vulcanizates was tested according to ASTM D573. Dumbbell-shaped samples were heated in a convection oven at 90°C for 7days. After aging, mechanical properties were measured. The aging coefficient (AG) was calculated to quantify the combined changes in tensile strength (TS) and elongation at break due to thermo-oxidative aging, relative to non-aged composites (Eq. 2).

2 $$AG=\frac{{TS}_{AA}\times {EB}_{AA}}{{TS}_{BA}\times {EB}_{BA}}$$

${TS}$ denotes tensile strength, while $EB$-stands for relative elongation at break. The properties are recorded both before (BA) and after ageing (AA).

Measurements of dielectric and electrical conductivity were conducted with a high-resolution broadband impedance analyzer (Schlumberger Solartron 1260). An alternating current electric field was applied covering a frequency spectrum of 0.1 Hz to 1 MHz. The sample holder was enclosed in electromagnetic shielding to mitigate low-frequency noise. Measurement automation was accomplished by a GPIB (IEEE 488) interface linking the analyzer to a personal computer, utilizing LabVIEW software for data gathering. Calibration procedures were executed before sample testing to mitigate stray capacitance effects. Measurement errors for permittivity (ε′) and loss tangent (tan δ) were assessed at 1–3%. Temperature regulation was achieved with a Pt100 sensor with an accuracy of ± 0.5 °C. Samples were preserved in desiccators with silica gel to inhibit moisture absorption and were then positioned in the measuring cell with P₂O₅ until measurements began.

## Results and discussion

### Hybrid characterization

#### XRF analysis and X-ray diffraction(XRD)

The XRF analysis was used to determine the composition of hybrids, Table 2 presents the XRF chemical analysis of the two hybrids. In this table it is shown that the two hybrids have high LOI values (62.11 & 45.55%) reflecting mostly the higher lignin content, it is appear that the hybrids are mostly composed of lignin. It can also be observed that the present of silica in Fe(LSF) is higher than Ni(LSF), also the percent of Fe_2_O_3_ is 15.9% and NiO is 39.46%.

**Table 2:** X-ray fluorescence (XRF) analysis of the Fe(LSF) and Ni(LSF) hybrids.

Main Constituents	Fe(LSF) Hybrid (wt%)	Ni(LSF) Hybrid (wt%)
SiO_2_	14.35	10.33
Fe_2_O_3_^tot^	15.90	0.02
NiO	0.006	39.46
Al_2_O_3_	0.05	0.06
CaO	0.09	0.14
Na_2_O	3.69	1.62
K_2_O	0.57	0.38
P_2_O_5_	0.14	0.07
SO_3_	0.23	2.20
Cl	2.71	0.17
LOI	62.11	45.55
MnO	0.060	–
CuO	0.024	–
ZnO	0.009	–
Cr_2_O_3_	0.014	–
Br	0.007	0.01
MgO	0.06	–

**Fig. 1:**
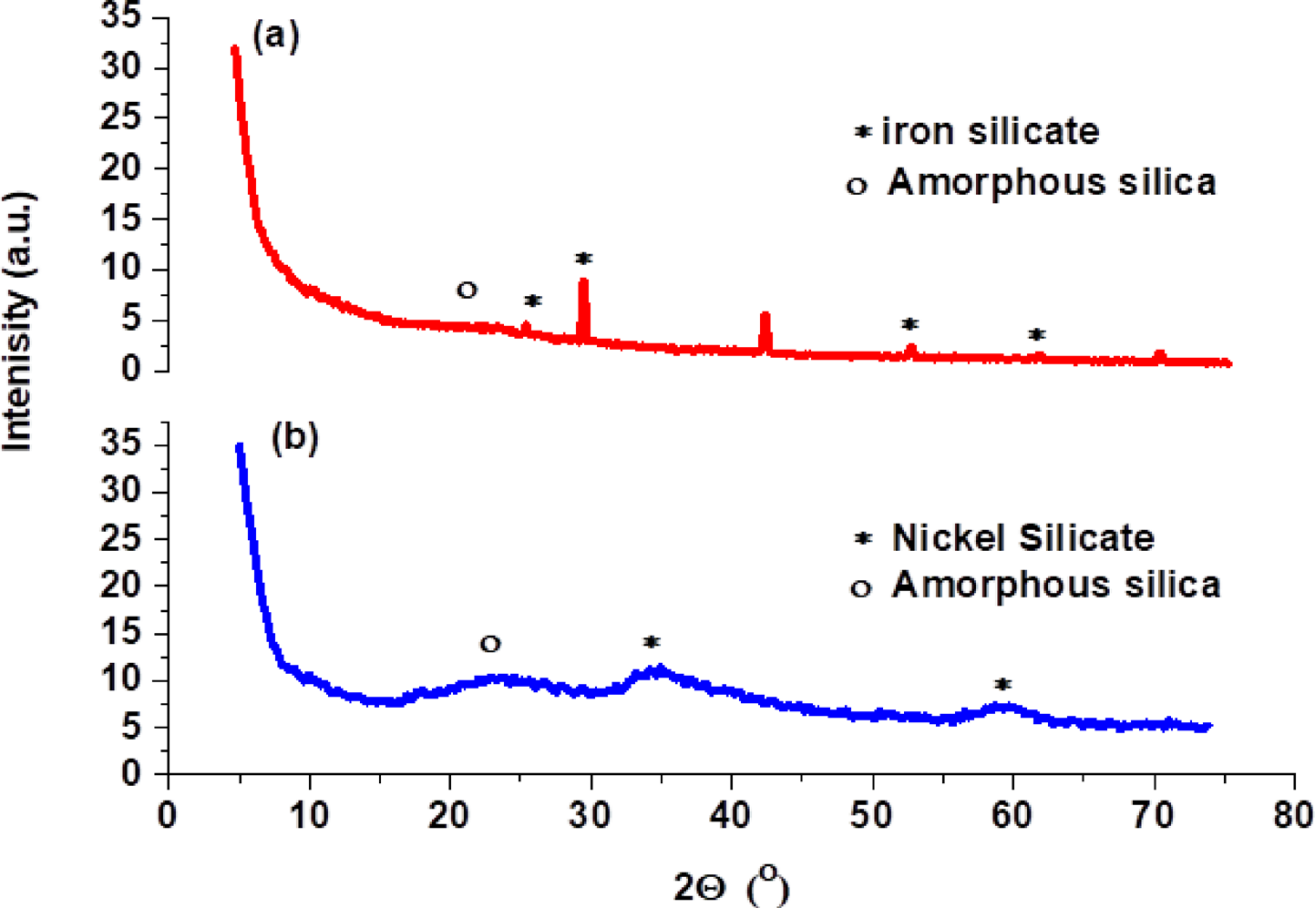
XRD of (**a**) Fe(LSF) and (**b**) Ni(LSF) hybrids.

As shown in Fig. 1, XRD was lead to define the phases of Fe(LSF) and Ni(LSF)hybrids by measuring resulting peak intensity as a function of the scattering angle (2θ) in degrees. In the case of Fe(LSF)hybrid Fig. 1a, the sharp peaks obtained indicate the presence of several crystalline phases. The crystallinity index (*CrI*) of the Fe(LSF) hybrid, calculated using the integration (area) method, was found to be 66.7%, indicating a predominantly crystalline structure with moderate amorphous content. The prominent diffraction peak at 26.97° corresponds well with JCPDS card no. 71-1667^27^. Similarly, the intense peaks observed at 31.32° and 56.04° are consistent with JCPDS card no. 01-076-0891^28^. The peak at 45.09° is likely attributable to trace amounts of elemental iron^27,29^. Additionally, the broad band spanning 5° to 20° suggests the presence of amorphous silica within the structure^30^.

In contrast, the X-ray diffraction pattern of the Ni(LSF) hybrid Fig. 1b demonstrate that it is primarily amorphous. In this case, the peak practical between 22° and 23° is likely due to amorphous silica, and the amorphous peaks seen at 35.41° and 60.01° indicate the presence of nickel silicate^31^. The crystalline phase of a material Fe(LSF)hybrid is associated with a lower density of defects as the complaint of the arrangement of (Fe) cations is less significant in comparison to the amorphous phase Ni(LSF) hybrid, therefore producing lower energy dissipation and lower dielectric loss^32^. In comparison, the disordered nature of the nickel (in the amorphous phase) produces irregular bonding environments which can aid the formation of dipoles and polarizations, producing higher dielectric constants^33^.

#### Morphological and structural characterization of hybrids

**Fig. 2:**
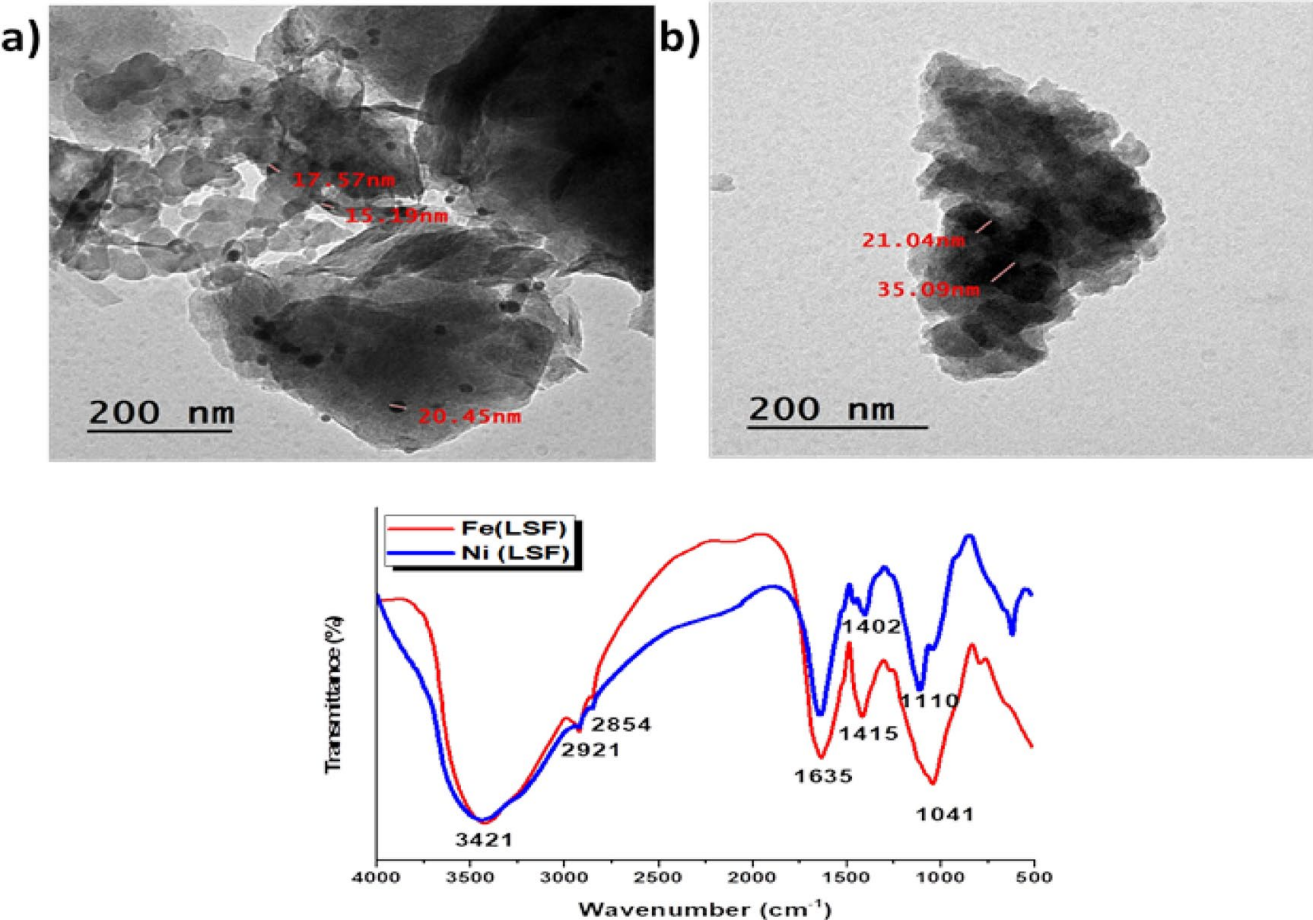
TEM micrographs and FTIR of (**a**) Fe(LSF) and (**b**) Ni(LSF).

TEM was used to determine the size, distribution, and structural characteristics of the prepared hybrids. Particle size is an important factor influencing their dispersion within a rubber matrix. As shown in the TEM images Fig. 2(a, b), both hybrids display a combination of dark, platelet-shaped lignin particles overlapping bright, spherical silica particles^22^. The particle dimensions obtained from TEM images are summarized in Table 3.

**Table 3:** Average particle dimensions and aspect ratios of Fe(LSF) and Ni(LSF) hybrid particles obtained from TEM images.

Sample	Average length (nm)	Average width (nm)	Aspect ratio (Length/Width)
Fe(LSF)hybrid	35.6 ± 16	29.5 ± 18	1.21 ± 0.9
Ni(LSF)hybrid	39.5 ± 9	39.5 ± 13	0.99 ± 0.7

The Fe(LSF) hybrid particles exhibited an average length of 35.6 ± 16 nm and a width of 29.5 ± 18 nm, while the Ni(LSF) hybrid particles showed an average length of 39.5 ± 9 nm and a width of 39.5 ± 13 nm. The corresponding aspect ratios were 1.21 ± 0.9 and 0.99 ± 0.7, respectively, indicating a nanoscale morphology with nearly isotropic particle shapes and uniform size distribution, which may facilitate homogeneous dispersion and strong interfacial interaction within the rubber matrix.

The FT-IR spectra showed in Fig. 2 for the Fe(LSF) and Ni(LSF) hybrids indicate characteristic absorption bands. The broad band appeared at 3421 cm⁻^1^ is for the O–H stretching vibrations associated with alcoholic and phenolic functionalities in lignin. The presence of bands at 2921 and 2854 cm⁻^1^ attributed to alkyl groups in both hybrids. The broad absorption observed at 1635 cm⁻^1^ is attributed to the C=O stretching of fatty acid moieties. Also the intense band at 1042 cm⁻^1^ indicates the Si–O stretching vibrations within SiO₂. The peak observed at 790 cm⁻^1^ is attributed to the O-Si–O vibrational mode of Silica.

**Fig. 3:**
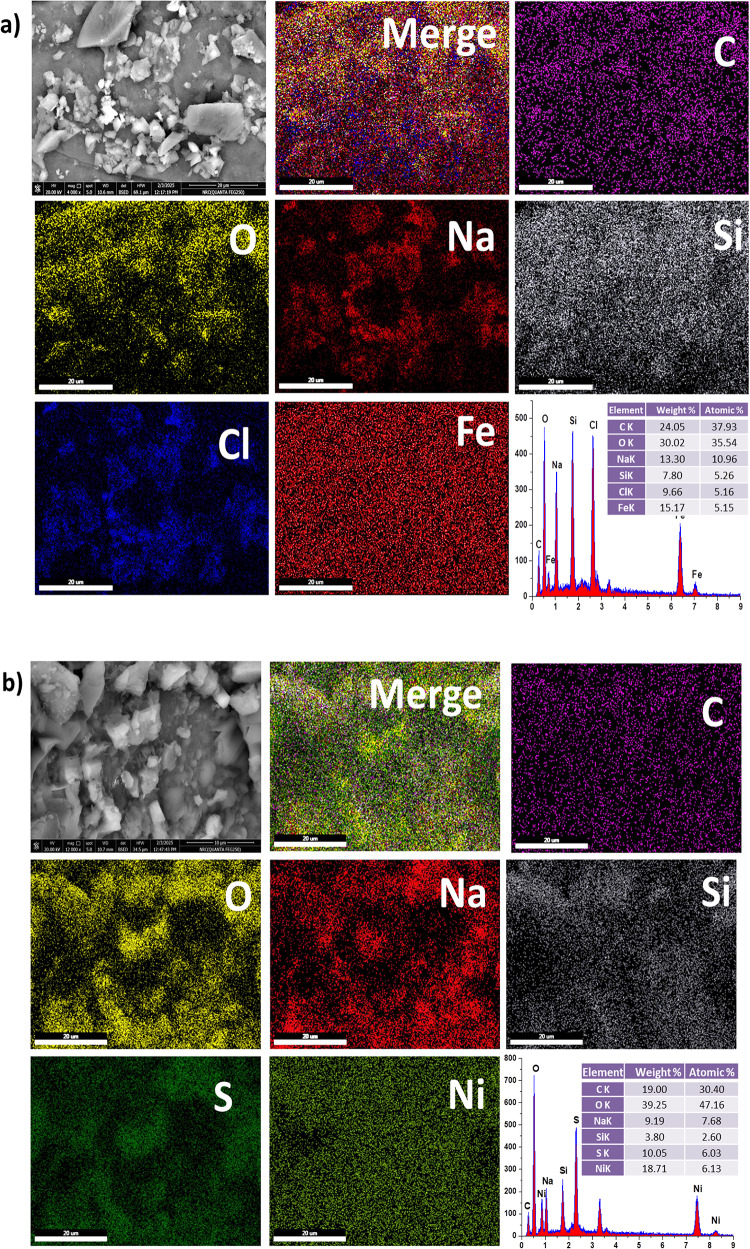
SEM, EDX, mapping of (**a**) Fe(LSF) and (**b**) Ni(LSF) hybrids.

Using SEM a technique that uses a focused electron beam to produce high-resolution images based on beam-sample interactions, the surface topography and composition of the hybrids were examined. The Fe(LSF) and Ni(LSF) hybrids’ SEM micrographs show that the silica, iron, and nickel clusters are properly distributed throughout the basic lignin matrix. The EDAX analysis showed that the organic content of the Fe(LSF) hybrid was C 24.05% wt. (representing the lignin and fatty acids), O 30.02% wt., Fe 15.17% wt., Si 7.8% wt., Cl 9.66% wt., and Na 13.3% wt. also the organic content of the Ni(LSF) hybrid was C 19.00% wt. (representing the lignin and fatty acids), O 39.25% wt., Ni 18.71% wt., Si 3.8% wt., S 10.05% wt., and Na 9.19% wt. Also, the EDAX mapping of the Fe(LSF) and Ni(LSF) hybrids show a homogenous distribution of atoms Fig. 3.

#### Dielectric properties of Fe(LSF) and Ni(LSF) hybrids

**Fig. 4:**
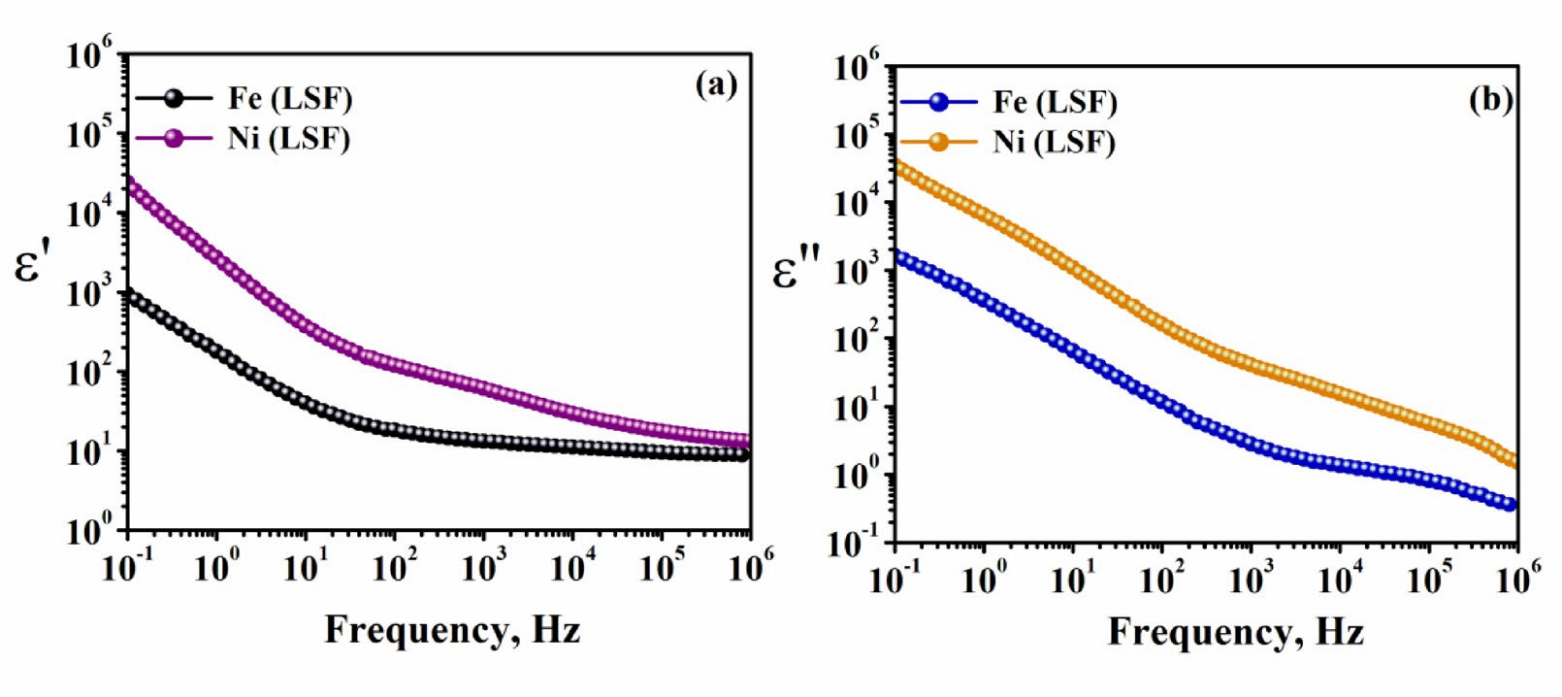
The variation of (**a**) the permittivity ε′ and (**b**) the dielectric loss loss ε″ with frequency at 30 °C for Fe(LSF) and Ni(LSF) hybrids versus the frequency of the applied field.

Figure 4a,b shows the frequency dependent permittivity ε′ and dielectric loss ε″ for Ni(LSF) and Fe(LSF) hybrids at 30°C, respectively which represents their function abilities in different conditions of applied electric fields. According to the XRF results in Table 2, the Ni(LSF) hybrid is mainly composed of NiO (39.46 wt%), while the Fe(LSF) hybrid contains considerably higher levels of FeO₃ (15.90 wt%) and SiO₂ (14.35 wt%). These hybrids are known to modify the electrical structure and polarization mechanisms inside the matrix, particularly in the presence of alternating electric fields^34–36^. The dielectric spectra Fig. 4 indicate also that Ni(LSF)hybrid exhibits higher ε′ and ε″ values over the frequency spectrum. This implies that, these composite exhibits greater space charge accumulation and interfacial polarization. The Maxwell–Wagner model posits that this phenomenon occurs due to the presence of semiconducting Ni^2^⁺ domains and insulating silica interfaces, which facilitate charge accumulation at grain boundaries^37^. Generally, the noticed significant decline in ε′ with increasing frequency supports the assumption that interfacial polarization predominates at low frequencies, whereas dipolar relaxation is more significant at high frequencies^38^.

Moreover, it is found that, Fe(LSF) hybrid exhibits a lower permittivity (ε′) when compared to Ni(LSF) hybrid, particularly within the low-frequency range. This indicates a constrained ability to polarize when an electric field is applied. Furthermore, it appears likely as a result of a lower percentage of Ni^2^⁺ and a higher concentration of Fe^**3+**^, which facilitates hopping conduction via Fe^**2+**^/Fe^**3+**^ redox couples. This approach aligns with the small polar on hopping theory, which posits that localized charge carriers influence AC conductivity σ and dielectric loss ε″^39,40^. In addition, this approach aligns with the XRD analysis that Fe(LSF)hybrid has crystalline phase structure which lower the dielectric loss, similarly, the higher LOI values (62.11 wt% for Fe(LSF) also indicated presence of a significant amount of organic and volatile matter. This may alter the dielectric relaxation by increasing free volume and segmental mobility^41^. Moreover, the trace amounts of Mn, Cu and Cr_**2**_ metal ions could act as dopants with slight modifications in the band structure leading to some localized states affecting dielectric dispersion^42^. The Fe(LSF) hybrid, which also contains a portion of SO_**3**_ and Cl may create ionic conducting pathways that could contribute to a rise in ε″ at low frequency. However, previous studies on ferrite systems reported that the Fe^3^⁺ behaves to produce large poling effect under AC field^43^, which is consistent with the strong dielectric response observed in hybrid material of Fe(LSF). Overall, the compositional discrepancy of Ni(LSF) and Fe(LSF) hybrids straight affect their dielectric response. Also the crystalline structure of Fe(LSF) hybrid and amorphous structure of Ni(LSF) hybrid which can influence the electrical properties of Ni(LSF) and Fe(LSF) hybrids making them suitable for advanced applications such as flexible electronics.

#### Conductivity and electric modulus of Fe(LSF) and Ni(LSF)hybrids

**Fig. 5:**
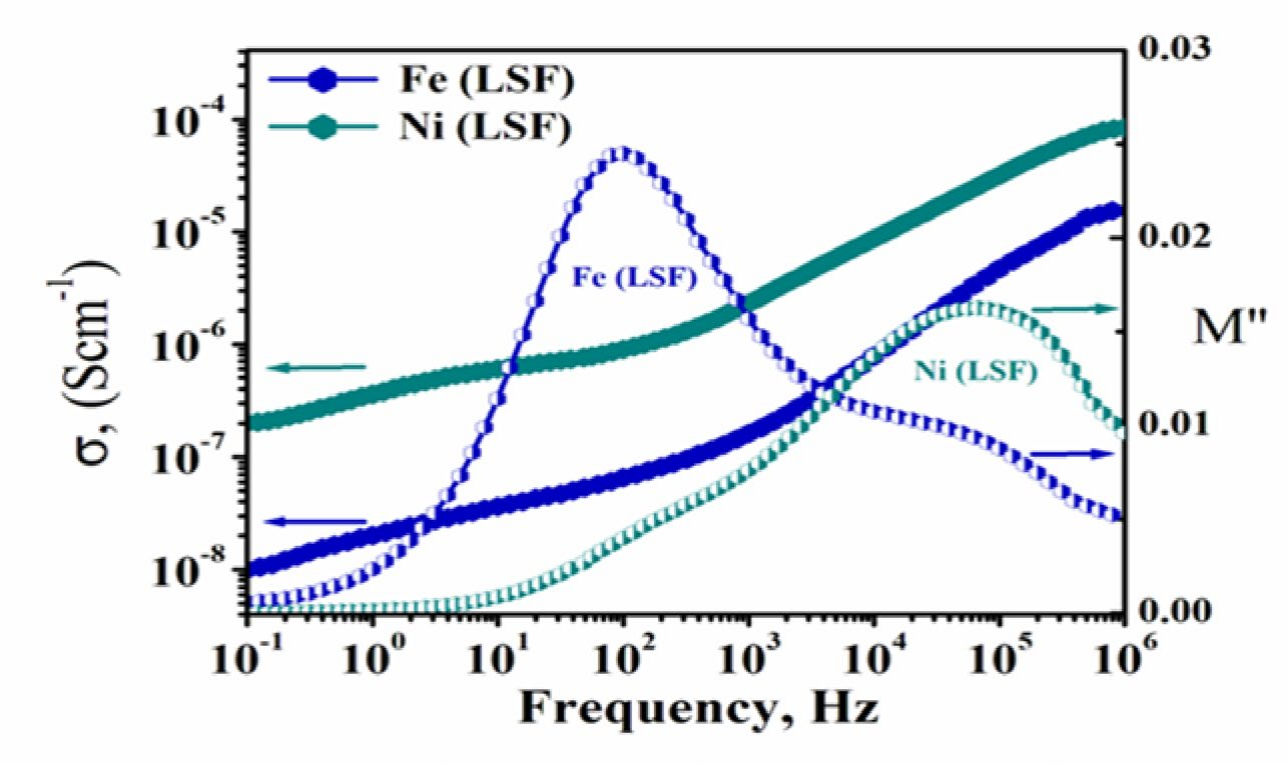
The ac-conductivity, σ (ω), and electric loss modulus, M″ versus frequency for of Fe(LSF) and Ni(LSF) hybrids at 30 °C.

Figure 5 depicts the frequency dependence of alternating current ac-conductivity (σ (ω)) and (b) electric loss modulus M″ for Fe(LSF) and Ni(LSF) hybrids at 30 °C. The ac-conductivity (σ (ω)) of Fe(LSF) and Ni(LSF) hybrids reveal frequency-dependent hopping transport, aligning with Jonscher’s power law^44^, expressed as:

3 $${\sigma }_{ac}\left(\omega \right)={\sigma }_{dc}+A{\omega }^{s}$$

where σ_dc_ represents the DC conductivity, A is a temperature-dependent constant, ω = 2πf denotes the angular frequency, and s is the frequency exponent (usually 0 < s < 1). The exponent *s* elucidates the fundamental conduction mechanism. The values of *s* are 0.55 for Ni(LSF) and 0.75 for Fe(LSF) hybrids respectively. The ac-conductivity is characterized at lower frequencies by a plateau-like behavior that yields the direct current dc-conductivity (σ_dc_) up to some characteristic frequency (fc). The deviation from plateau at very low frequency is due to electrode polarization effects^45^. The conductivity trend of the Fe(LSF) hybrid suggests a transition from localized hopping to long-range conduction and aligns with the small polar on hopping theory, which posits that charge carriers transition between Fe^**2+**^ and Fe^**3+**^ centers^39^. Conversely, Ni(LSF) hybrid displays a consistent increase in conductivity during the whole frequency spectrum, representing a more continuous conduction pathway. P-type conduction arises through Ni^2^⁺ vacancies, assisting hole mobility inside the oxygen 2p band. The refined σ -profile shows correlated barrier hopping (CBH) and defect-assisted conduction, predominant in systems abundant Ni(LSF) hybrid^46^. Otherwise, the imaginary constituent of the electric modulus (M″) provides additional insights into relaxation processes. Fe(LSF) has a peak in M" at approximately 10^**3**^ Hz, indicative of the characteristic relaxation time for hopping carriers. The broader peak reflects a dispersion of relaxation times caused by the presence of various oxide constituents and structural defects^46^. Ni(LSF) hybrid reveals its M" peak at a raised frequency (~ 10^4^ Hz), representing that the relaxation processes occur further rapidly and the interfacial impedance is diminished. This alteration indicates that Ni^2^⁺ domains enhance charge mobility and reduce relaxation periods, consistent with Maxwell–Wagner-Sillar polarization effects^41^. The disparity in dielectric relaxation and conductivity profiles between the two hybrids arises from the metals constituting them. As previously mentioned, Ni(LSF) and Fe(LSF) hybrids are affecting both carrier type and mobility. The incorporation of insulating silica and minor metals further alters the dielectric environment by generating localized states and interfacial barriers^47^.

### Vulcanization properties of EPDM composites

#### Curing characteristics

**Fig. 6:**
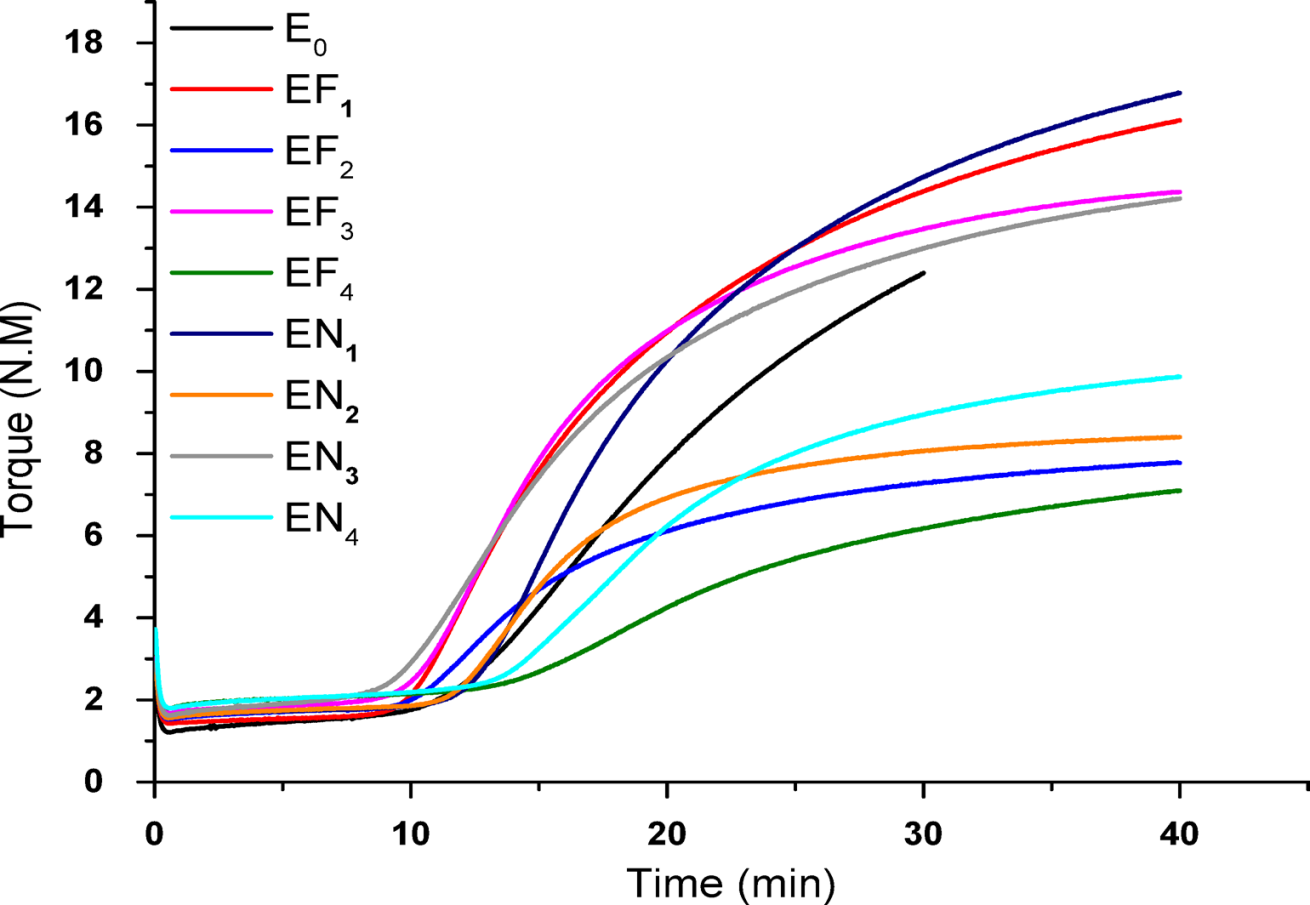
Vulcanization curves for EPDM composites incorporating various proportions of Fe(LSF) and Ni(LSF) hybrids.

Figure 6 demonstrates the torque-time curves during vulcanization for EPDM composites incorporating various loadings of Fe(LSF) and Ni(LSF) hybrids. For comparative purposes, the specific parameters of all samples are comprehensively listed in Table 4. The rheographs indicate that all vulcanizates display similar cure curve profiles when Fe(LSF) and Ni(LSF) hybrids are employed as activator systems. After attaining the minimum torque, the curves show a gradual increase to a peak value, followed by either a plateau or slight decline with continued vulcanization time^48^. Table 4 demonstrates that substituting ZnO partially with Fe(LSF) and Ni(LSF) hybrid led to a significant rise in the maximum torque (M_**H**_) minimum torque difference (ΔM) of EPDM composites. Nevertheless, further incorporation of the hybrids to replace both ZnO and/or stearic acid resulted in only a marginal increase in M_**H**_ and ΔM. An increase in hybrids content led to a corresponding rise in the minimum torque (M_**L**_), reflecting changes in the material’s processability. At higher contents of Fe(LSF) and Ni(LSF) hybrids, the deterioration of the crosslinking network weakened the interfacial bonding with the EPDM matrix, causing an increase in M_**L**_ and a decrease in M_**H**_ and ΔM. With the partial substitution of ZnO and full replacement of ZnO and/or stearic acid, both scorching time (T_**S2**_) and optimum cure time (T_**C90**_) declined, accompanied by an increase in CRI, suggesting a more rapid vulcanization process^49,50^.

**Table 4:** Curing data of different EPDM composite materials.

Samples Codes	E0	EF1	EF2	EF3	EF4	EN1	EN2	EN3	EN4
M_H_ ( dNm)	14.57	16.12	7.79	14.37	7.1	16.79	8.4	14.21	9.88
M_L_ (dNm)	1.36	1.42	1.52	1.67	1.79	1.57	1.55	1.62	1.79
∆M (dNm)	13.21	14.7	6.27	12.7	5.31	15.22	6.85	12.59	5.09
ts_2_ (min)	16.48	11.29	12.79	11.4	18.59	13.63	13.56	10.88	15.9
t_c90_ (min)	34.66	31.15	28.35	27.96	33.49	32	25.42	29.72	30.89
CRI, %	5.5	5.035	6.422	6.14	6.711	5.44	8.43	5.31	6.671
Tanδ	0.0158	0.0293	0.0488	0.0236	0.0622	0.0101	0.0463	0.0386	0.0751

 The fact that the reduced T_**C90**_ of the Ni(LSF)/EPDM composite was lower than those of Fe(LSF) and ZnO systems indicates that the latter had a lower vulcanization efficiency. The mentioned improved was through the combination of van der Waals and electrostatic interactions at the Ni(LSF)–EPDM interface. These two forces not only contribute to the even distribution of the Ni(LSF) hybrid but also ensure strong adhesion to the EPDM chains, thus reducing particle agglomeration and granting better dispersal of stress. The bonding of Ni²⁺ ions through electrostatic means to the mildly polarized EPDM backbone also confines the chain mobility in the interface which in turn becomes a more reactive site for sulfur crosslinking thus, leading to the enhanced creation of the linkages. In other words, the interactions that occurred at the interface accelerated the curing process as well as the overall efficiency of crosslinking, these observations are consistent with^51,52^. The Tanδ of EF_4_ and EN_4_ composites represented the high value in Table 4, because the higher Tanδ could responsive to viscous behavior and internal molecular friction under mechanical stress leading to more energy dissipation.

#### Mechanical properties

**Fig. 7:**
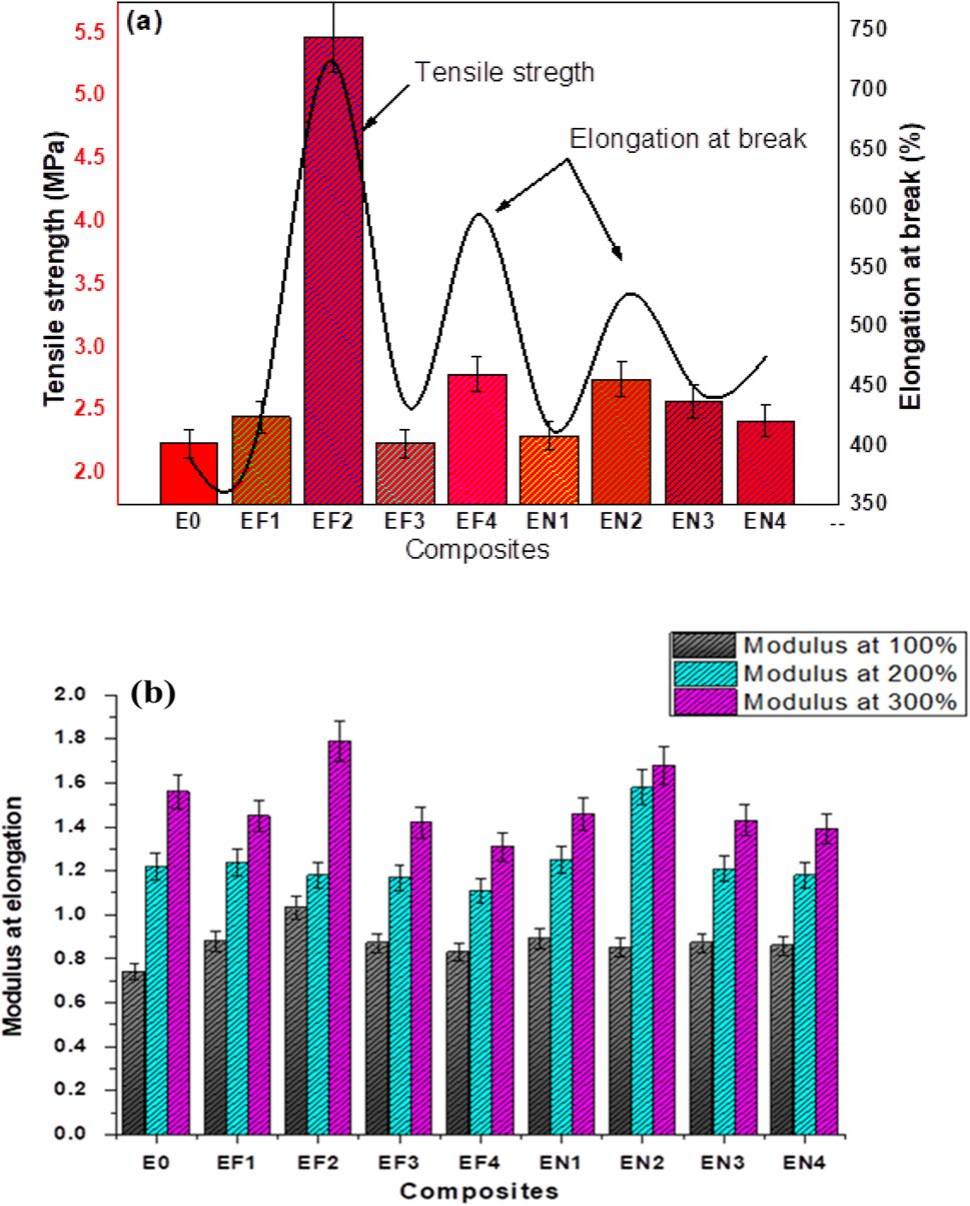
Mechanical properties of EPDM vulcanizates, including (**a**) tensile strength and elongation at break %, and (**b**) modulus at 100,200 and 300% elongation.

To assess the mechanical properties of EPDM composites, static mechanical tests were conducted using a universal tensile testing machine. The partial replacement of ZnO and/or replace both ZnO and/or stearic acid lead to improve tensile strength, elongation at break, and imparts mechanical strength in the EPDM composites. Figure 7 illustrates the mechanical behavior of EPDM composites as a function of Fe(LSF) and Ni(LSF) hybrids loading. The results indicate that tensile strength (T.S) of Fe(LSF)/EPDM and Ni(LSF)/EPDM composites are greater than those of ZnO/St. acid/EPDM composites. The improvement is due to the large specific surface area, and effective dispersion of hybrids in the EPDM matrix which exhibits excellent mechanical properties. With the inclusion of 5 phr Fe(LSF) and Ni(LSF) hybrids to completely substitution of ZnO, the tensile strength of both composites reach 5.57 MPa and 2.75 MPa, respectively, reflecting enhancements of 148% and 23% compared to blank composites with ZnO (2.24 MPa). Interestingly, it can be noted that Fe(LSF) hybrid as a cure activator can achieve the best strength value compared to the other composites. It is not possible to prove that all the Fe(LSF) hybrid has been consumed during the vulcanization reaction, some amount might have acted as a reinforcing system indirectly to increase the performance of the adhesive^53^. The T.S start declining by replacing ZnO and stearic acid with Fe(LSF) and Ni(LSF) hybrids at 7 phr. This decline may occur due to agglomeration which weakens the composites rather than reinforcing them.

It was observed that from Fig. 7a E0 composites has strain of about 390%. After partially and/or completely replacing of activators system, the elongation at break increases up to 725% (EF_**3**_) and 525% (EN_**2**_) due to better distribution of Fe(LSF) and Ni(LSF) particles within EPDM matrix and reduces the risk of crack propagation, allowing EPDM composites to stretch more before breaking. There is slight reduction in elongation at break due to agglomeration of hybrids particles which restricts the movement of the matrix. The specific modulus such as M100, M200 and M300 in Fig. 7b, c has similar trends for all the vulcanizates. The mechanical properties indicated that the conventional activator system (ZnO and St. acid) could be efficiently replaced by green Fe(LSF) and Ni(LSF) hybrids, and used in different rubber applications^23,53^. The reinforcement of rubber composites through activators is attributed to strong interfacial bonding from chemical (covalent/ionic), physical (van der Waals, hydrogen bonding, adsorption), and mechanical (chain inter-diffusion and entanglement) interactions, leading to improved stress transfer and mechanical properties.

#### Thermo-oxidative ageing influence

EPDM rubber is known for being used in outdoor applications due to its ability to withstand the effects of external factors which cause aging and loss of performance properties over time. Of these one important factor is thermo-oxidative aging. In this study, an attempt was made to investigate the influence of two new alternative vulcanization activators Fe(LSF) and Ni(LSF) hybrids on the thermo-oxidative aging resistance of EPDM. Composites were aged for 7 days at 90 °C, followed by evaluation of their mechanical properties and compared to the non-aged samples. The findings are presented in Table 5. The tensile strength after aging was very much reduced when the conventional activator system (ZnO and stearic acid) were partially replaced with certain concentrations of Fe(LSF) and Ni(LSF) hybrids (formulations EF_1_ & EN_**1**_). EF_1_ exhibited a reduction of 14.68%, while EN_**1**_ reduced by 15.22%. More replacement of ZnO with the prepared hybrids (formulations EF_**2**_ and EN_**2**_) resulted in even better reductions: 48.8% for EF_**2**_ and 22.9% for EN_**2**_. In formulations without stearic acid, sample EF_**3**_ exhibited a 13.8% decline in tensile strength, while EN_3_ showed a smaller reduction of 8.17%. Additionally, Table 5 revealed that the decrease in elongation at break was less pronounced in Ni(LSF)/EPDM composites compared to Fe(LSF)/EPDM composites. Once Fe(LSF) and Ni(LSF) hybrids were used as the sole activators, EF_**4**_ indicated a 34.1% reduction in tensile strength, but EN_**4**_ established only a 4.1% decline.

**Table 5:** The impact of thermo-oxidative ageing on the tested properties of EPDM composites.

Samples Codes	Ageing	E_0_	EF_1_	EF_2_	EF_3_	EF_4_	EN_1_	EN_2_	EN_3_	EN_4_
Tensile Strength, MPa	before	2.24	2.45	5.47	2.24	2.79	2.3	2.75	2.57	2.42
	after	1.36	2.09	2.8	1.93	1.84	1.95	2.12	2.36	2.32
Elongation at break, %	before	390	425	725	435	595	415	525	450	475
	after	380	390	545	410	440	395	460	425	440

**Fig. 8:**
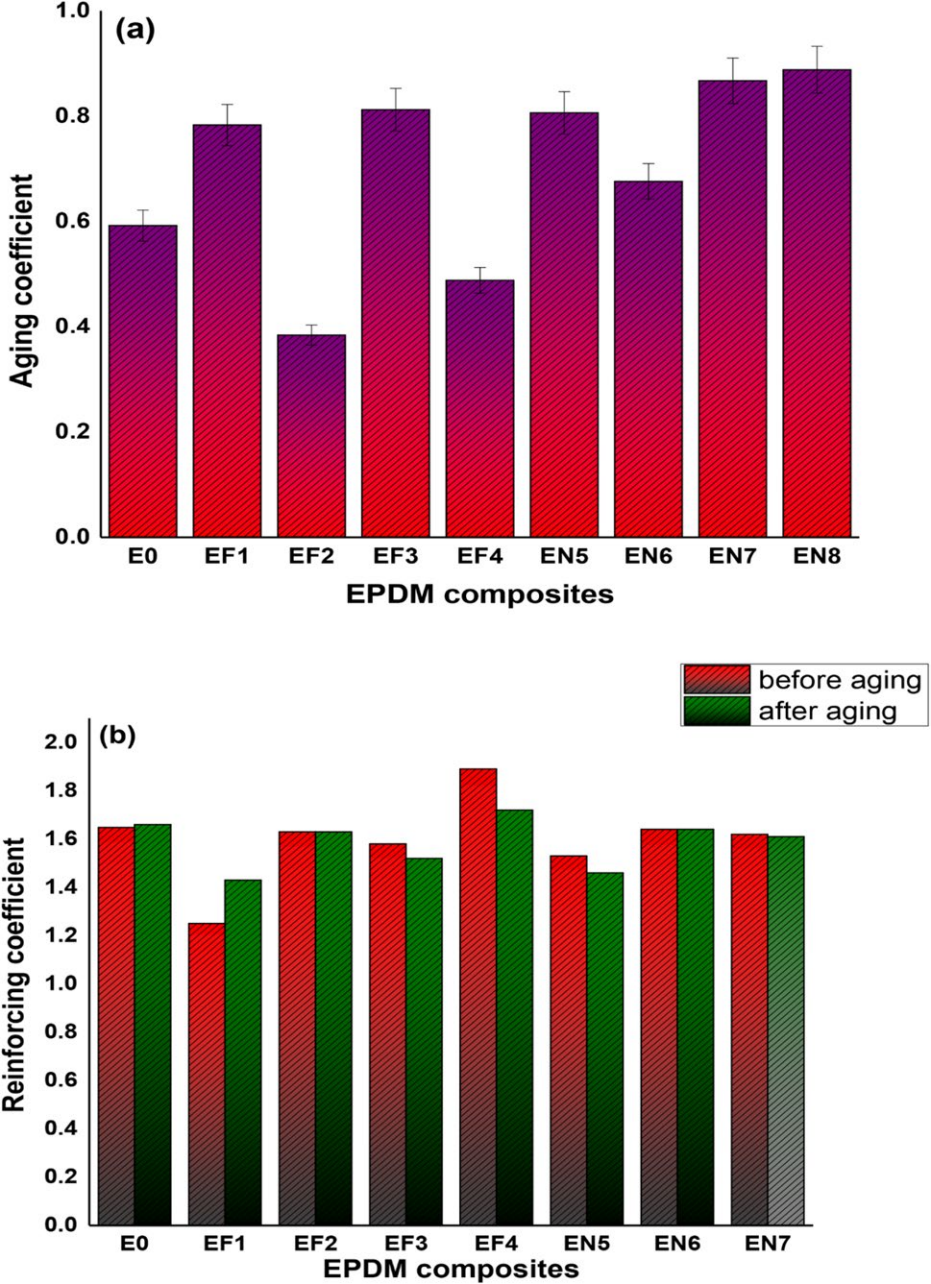
Aging behavior of EPDM vulcanizates (90 °C × 7 days) showing (**a**) aging coefficient and (**b**) reinforcement index.

The present research investigated the viability of substituting conventional ZnO activators by prepared Fe(LSF) and Ni(LSF) hybrids, with an emphasis their thermal behavior. The findings reveal that the Ni(LSF) hybrid is a more promising and effective substitute to ZnO compared to the Fe(LSF) hybrid. As presented in Table 5, the incorporation of Ni(LSF) hybrid led to considerably enhanced in the mechanical properties and thermal stability of EPDM composites after aging^54^. Analysis of the aging coefficient (AG) further confirms the superior performance of Ni(LSF)/EPDM composites. As observed in Fig. 8, Ni(LSF)/EPDM composites attained higher AG values than both the control and Fe(LSF)/EPDM composites, reflecting the better retention of mechanical properties after aging. Fe(LSF)/EPDM composites exhibited lower AG values at lower retention of mechanical properties after aging. Higher AG values generally reflect improved structural stability and lower degradation rates during aging, suggesting that Ni(LSF) composites possess superior resistant to thermal aging compared to the Fe(LSF) composites^55^.

The enhanced thermal resistance observed in Ni(LSF)/EPDM composites compared to Fe(LSF)/EPDM composites, despite both hybrids containing silica and thus silanol groups, can be attributed to the stronger coordination ability of Ni^2^⁺ ions with oxygen donor groups such as silanol (Si–OH) and phenolic groups, compared to Fe^3^⁺. This stronger interaction promotes better dispersion of silica within the rubber matrix and enhances interfacial bonding, which results in greater improvements in conductivity and mechanical stability. In contrast, Fe^3^⁺ ions preferentially undergo hydrolysis and tend to form aggregated phases, which limit their ability to interact with silanol groups as effectively as Ni^2^⁺^56^.

This results in less deterioration of mechanical properties (TS and elongation at break) compared to vulcanizates containing zinc oxides (EF_**3**_ and EN_**3**_). These findings are consistent with those reported by^52,53^. Reinforcing Index (RI) is used to evaluate the effectiveness of activator in polymeric materials. However, the reinforcing index (RI) serves as a valuable metric for assessing the effectiveness of an activator in a polymeric material. Specifically, it quantifies the contribution of a material or activation method to the strength and other desirable properties of the composite material^23,57^. The reinforcing index is well-defined as the ratio between the modulus at 300% elongation (M_**300**_) and the modulus at 100% elongation (M_**100**_), as showed in Eq. (3):

4 $$RI=\frac{{M}_{300}}{{M}_{100}}$$

Figure 8 illustrates an increasing trend in the reinforcing index for samples loaded with Ni (LSF) hybrid compared to those with Fe (LSF) hybrid, both before and after aging^57,58^. Notably, replacing stearic acid with the prepared composites had no significant effect on the RI, as the values remained approximately the same for Fe(LSF) and Ni(LSF) hybrids before and after aging. However, the reinforcing index for Ni(LSF) hybrid was consistently higher than that for Fe (LSF) hybrid when used as an activator system. Reinforcement is typically enhanced through strong interfacial bonding, either physical or chemical, between the activator and the EPDM rubber matrix. The mechanical properties enhanced owing to this bonding which enhances effective stress transfer^23^. This bonding improved the mechanical characteristics and increased the efficiency of stress transfer. Figure 8 clearly demonstrates that Ni (LSF) outperforms Fe (LSF) as an activator for EPDM rubber. One key advantage of combining Ni (LSF) with the EPDM rubber matrix is the strong adhesion and contact achieved between the nonpolar rubber and the polar activator^59^.

#### FTIR analysis

**Fig. 9:**
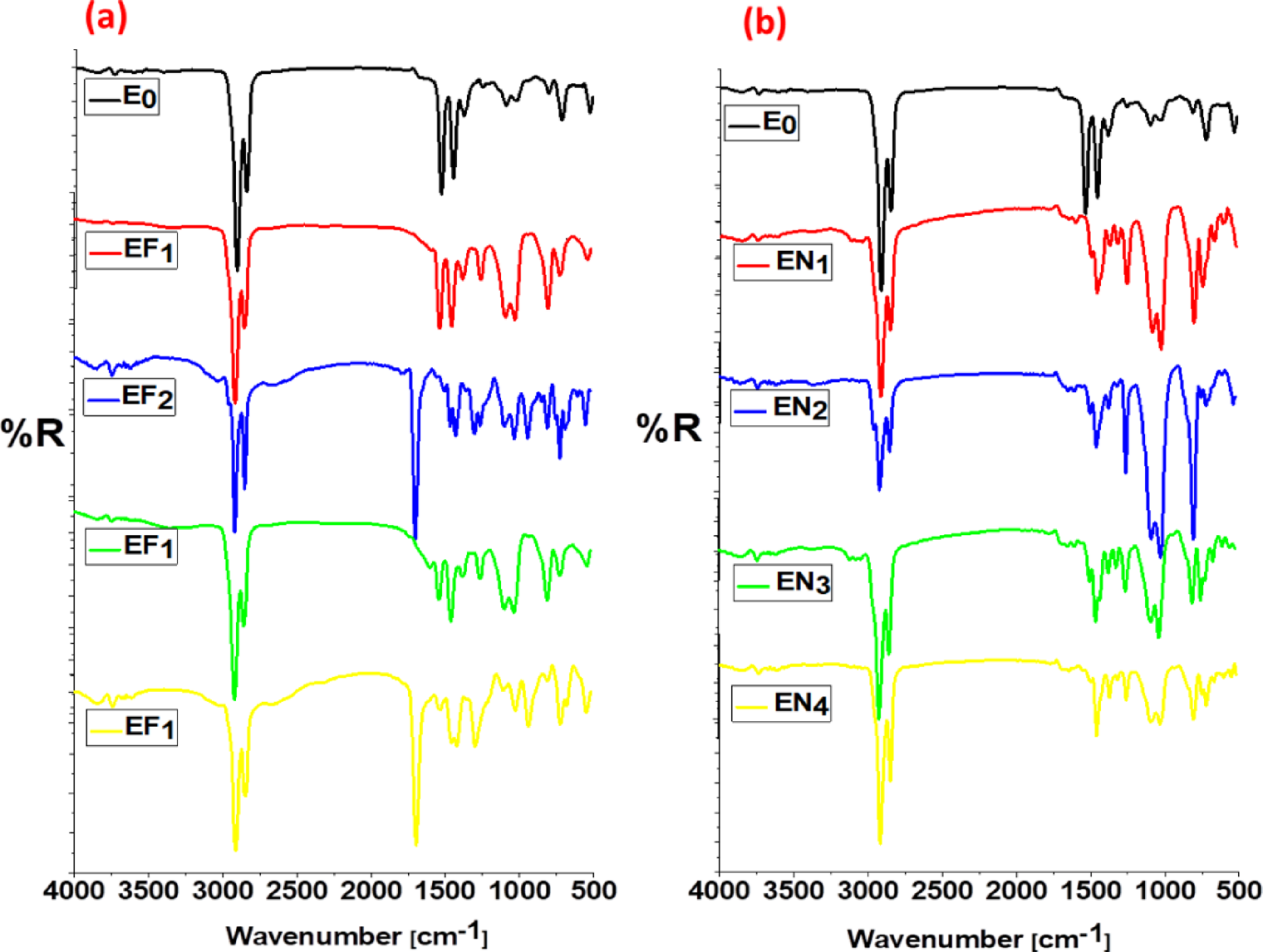
FTIR spectra of EPDM vulcanizates incorporating various proportions of (**a**) Fe(LSF) and (**b**) Ni(LSF) hybrids.

Figure 9 illustrates that Fe(LSF) and Ni(LSF) hybrids effectively replace the conventional ZnO-stearic acid activator system in EPDM composites. E₀ composite exhibits characteristic EPDM bands appear at 2918 and 2850 cm⁻^1^ (–CH_2_ stretching), along with 1465 cm⁻^1^ for –CH₂ bending, and 1050 cm⁻^1^ for C–O stretching, in addition to a weak Zn–O absorption around 550 cm⁻^1^. Upon partial and complete substitution of ZnO with Fe(LSF) (EF₁–EF₄) and Ni(LSF) (EN₁–EN₄), distinct spectrum changes are observed. The existence of new Fe–O and Ni–O bands within 520–570 cm⁻^1^ and disappearance of Zn–O vibrations confirm the effective incorporation of these metal hybrids in the EPDM matrix during vulcanization^60^. Additional observed shifts in C–O and C = C regions (1600–1000 cm^**-1**^), together with the broad –OH stretching bands around 3400 cm^**-1**^ indicate strong interfacial interactions between the metal centers and the EPDM matrix. These spectral variations verify that the metal hybrids participate in the vulcanization process by formation of metal-sulfur complexes similar to those formed by ZnO. thereby enhancing crosslink density, and confirms their efficacy as effective, eco-friendly activators for ZnO due to the catalytic activity of Fe(LSF) and Ni(LSF) hybrids during sulfur curing. Enhanced intensity at 1050 cm^**-**1^ (C–O stretching) for EF_**2**_ and EN_**2**_ suggests increased crosslink formation and stronger interfacial bonding^61^.

Overall, FTIR confirms that the Fe(LSF) and Ni(LSF) hybrids are act as effective and green ZnO substitutes and improved curing performance in EPDM composites.

#### Morphological studies

**Fig. 10:**
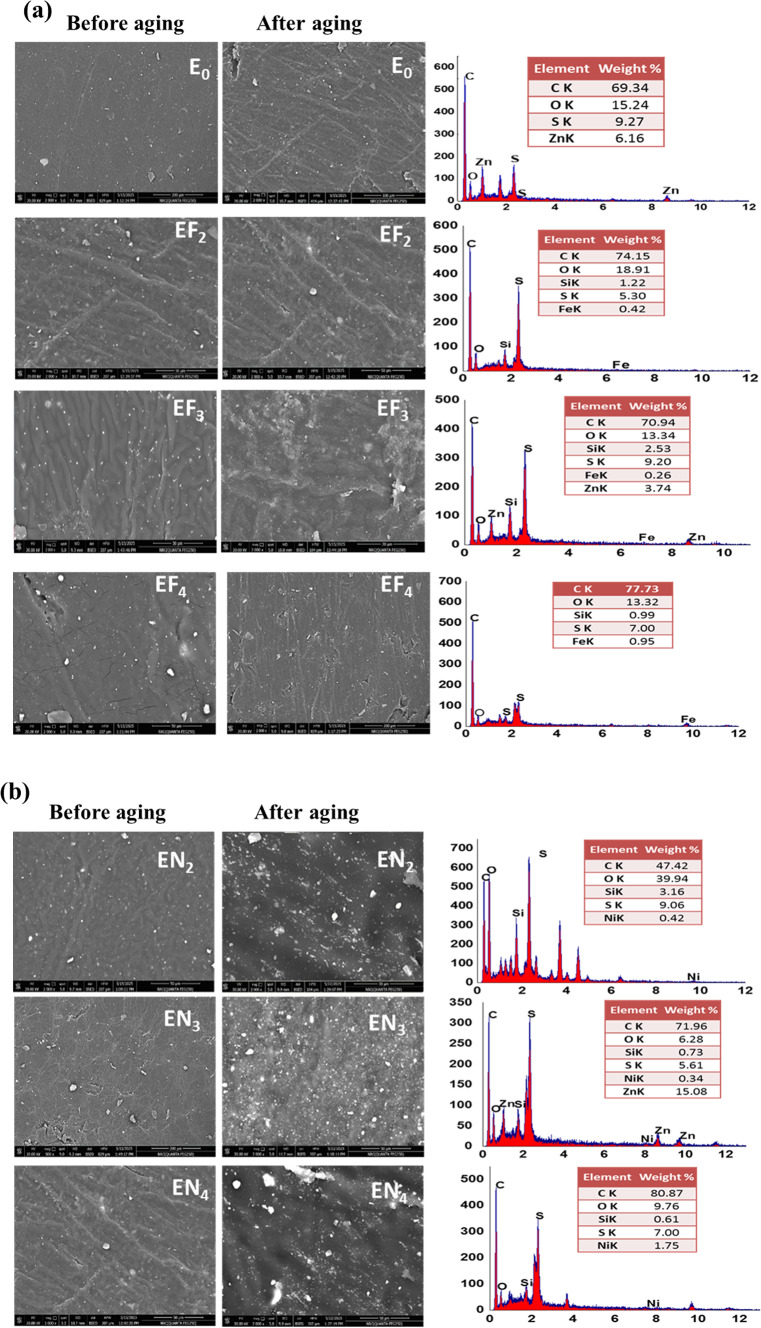
SEM micrographs and EDX spectra of EPDM vulcanizates before aging and after aging (aging condition: 90 °C × 7days) of (**a**) Fe(LSF) and (**b**) Ni(LSF) hybrids.

Figure 10 shows SEM images along with EDX analysis. This highlights the elemental distribution and fracture surfaces for EPDM composites with Fe(LSF) and Ni(LSF) hybrids as curing activators. The structure of neat EPDM composites (E_**0**_) shows a stable and uniform distribution of components. The identification of C, O, Zn, and S in E_0_ through EDX analysis confirms effective vulcanization and even distribution of the formulation components. As shown in Fig. 10a, using Fe(LSF) and Ni(LSF) hybrids instead of ZnO and/or stearic acid results in a rougher surface. This roughness suggests good homogeneity and effective dispersion of the eco-friendly additives. This is likely due to improved interfacial adhesion and smaller sizes of the dispersed phase domains. The well-dispersed hybrids promote stable physical interaction between EPDM chains and residual vulcanization byproducts (unreacted sulfur, zinc compounds (ZnS), accelerator residues, etc.). Oils, antioxidants, and plasticizers may also remain. This good dispersion serves to maintain this relationship and thus enhances the mechanical properties^13^.

As cited earlier, stearic acid plays a key role in the activation process by promoting the dispersibility and solubility of ZnO in the elastomer phase, favoring reactivity, so that presence of St. acid and the fatty acid in the EF_**2**_ and EN_**2**_ samples was partially replaced ZnO likely helped reduce cross-linking density, correlated with the results seen in Table 4. Further analysis of the surface morphology in EF_**4**_ and EN_**4**_ reveals smaller pores and the presence of agglomerates of larger particles, which could probably be due to non-uniform deposition of the hybrid. The elemental composition of Fe(LSF)/EPDM and Ni(LSF)/EPDM composites, as determined by EDX analysis, revealed the presence of C, O, and S as primary elements of the EPDM matrix, along with Si, which likely originates from silica present in the incorporated hybrids. Compared to ZnO/St. acid/EPDM composites, the increased content of O and C in the Fe(LSF)- and Ni(LSF)-based systems supports the presence of lignin and fatty acid constituents in the hybrids. Moreover, Fe and Ni were detected in their respective hybrid formulations. SEM images (Fig. 10) revealed that the green alternatives, Fe(LSF) and Ni(LSF) hybrids used as activators, produced comparable phase morphologies in the final vulcanized EPDM materials^14,62^. To assess the effects of thermal aging, SEM was used to analyze the morphology of EPDM composites formulated with green activators replacing the standard activator system after 7days at 90 °C.

SEM images in Fig. 10 reveal no significant morphological differences in the EPDM composites before and after thermal aging. According to Fig. 9, the surfaces of E_**0**_, EF_**2**_, EF_**3**_, and EF_**4**_ composites retained their smooth morphology after thermal aging, with slight surface cracking and unchanged surface coloration. After thermal aging, the fracture surfaces of EN_**2**_, EN3, and EN_4_ composites became more irregular and coarser^63^. Similarly, the Ni(LSF)/EPDM composites exhibited a denser structural formation and greater surface roughness relative to Fe(LSF)/EPDM and ZnO/Stearic acid/EPDM composites. The observed phenomenon can be attributed to the free radical capturing ability of Ni(LSF) hybrid, which strengthens its interaction with the EPDM matrix and leads to a rougher, more compact fracture surface. These findings are in good agreement with those reported in study^64^.

TEM and SEM analyses showed that both hybrids contained overlapping morphology of nanoparticles. Smaller sized particles increase the dispensability and available interfacial area for polymer interactions in the EPDM matrix. Better dispensability leads to more efficient stress transfer and reinforcement. In addition, nano-particles can also serve as active sites during curing, promoting cross-linking and network formation, particularly in Ni(LSF) composites with stronger interfacial interactions.

#### Dielectric properties of EPDM composites

**Fig. 11:**
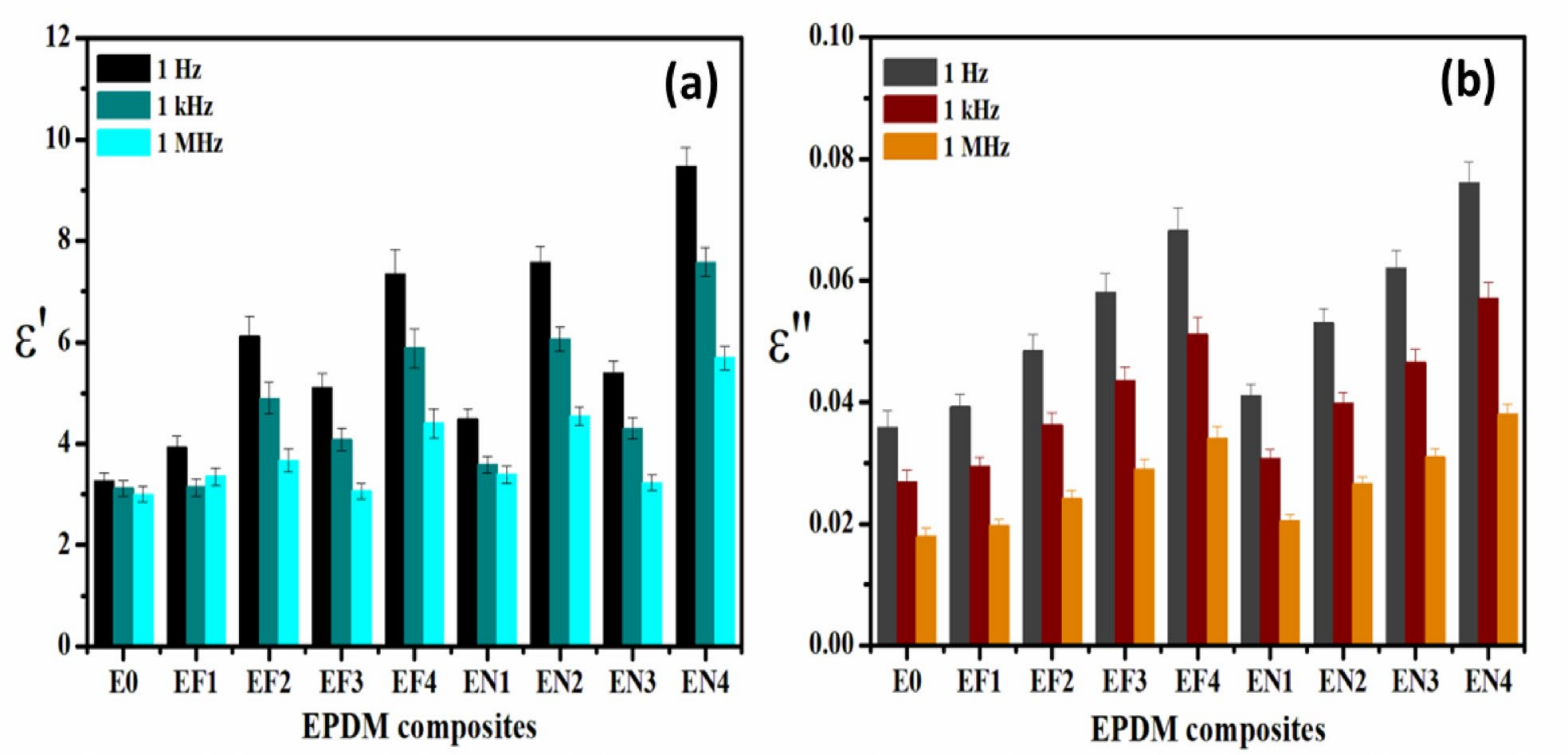
The variation of (**a**) the permittivity ε′ and (**b**) the dielectric loss loss ε″ at 30 °C for EPDM loaded with different concentration of Fe(LSF) and Ni(LSF).

The dielectric response of EPDM composites was systematically investigated across a frequency range of 1 Hz to 1 MHz. The data are summarized at three frequencies covering the whole range for ease comparison and presented in Fig. 11. This figure illustrates the variation in both the real (ε′) and imaginary (ε′′) components of the hybrid permittivity for the neat EPDM (E_**0**_), as well as the EN- and EF-series filled composites.

#### Permittivity (ε′) of EPDM composites

The variation of permittivity (ε′) of EPDM composites at different frequencies is depicted in Fig. 11. At 1 Hz, the composites exhibit elevated ε′ values, with EN_**4**_ attaining a maximum near 11. This enhancement is attributed to interfacial polarization, where charge accumulation at the activitor–matrix interfaces leads to the observed low-frequency response. The decrease in ε′ at 1 kHz and 1 MHz indicates that dipolar entities and space charges are unable to respond to the rapidly fluctuating field, consistent with the Maxwell–Wagner-Sillar model^65^. However, it is clear that, the EN-series composites (containing Ni-based activitor) typically exhibit a higher ε′ than the EF-series (containing Fe-based activator). This indicates that Ni^2^⁺-rich domains induce greater space charge polarization due to their semiconductor nature and defect-laden interfaces^66^.

#### Dielectric Loss (ε") of EPDM composites

Figure 11a,b illustrates the fluctuation of dielectric Loss (ε") of EPDM composites at various frequencies. The ε″ values diminish in a frequency-dependent manner, with EN_**3**_ demonstrating the most loss at 1 Hz (about 0.08). This indicates that significant energy is dissipated by dipolar relaxation and ionic conduction. The decline in ε″ at elevated frequencies indicates a reduction in charge migration speed and interfacial friction, consistent with Jonscher’s universal dielectric response^38^. Moreover, the elevated ε″ in Ni-based hybrid may result from Ni^**2+**^ vacancies and oxygen-related defects, which facilitate hopping conduction and enhance dielectric relaxation^41^. Conversely, Fe-based hybrid exhibits reduced ε″, consistent with their superior insulating characteristics and diminished carrier mobility^67^.

Generally, the dielectric properties of EPDM composites entail a complicated interplay between polarization induced by activators and dispersion influenced by additives. When used in moderate quantities (such as EF_**1**_, EF_**3**_, EN_**2**_, and EN_3_), ZnO mitigates low-frequency dielectric losses by inhibiting interfacial polarization. This is likely due to the superior dispersion of the activator hybrids as revealed by SEM (Fig. 9a) and a reduced accumulation of space charge. The stabilizing effect reduces the ε″ values within the 1 Hz to 1 kHz spectrum, indicating a restriction in dipolar relaxation. Conversely, Stearic acid promotes significant polarization activity, particularly in composites containing Fe(LSF) and Ni(LSF) hybrids (EF_**2**_, EF_4_, EN_**1**_, EN_**2**_), where elevated ε′ and ε″ values indicate enhanced dipole reorientation and relaxation processes^68^. The synergistic effects of stearic acid and Ni(LSF) hybrid, as seen in EN6, further enhance dielectric dissipation, hence facilitating greater dipolar contributions and improved matrix mobility. The results indicate that ZnO serves two significant functions: it diminishes space-charge polarization, while stearic acid enhances the interaction between activators and the matrix. Both factors are crucial for tailoring the dielectric response of multifunctional EPDM systems. The obtained results suggest that by adjusting the activator type and concentration, the dielectric response of EPDM composites can be successfully optimized for various applications^69,70^.

#### Conductivity analysis of EPDM composites

**Fig. 12:**
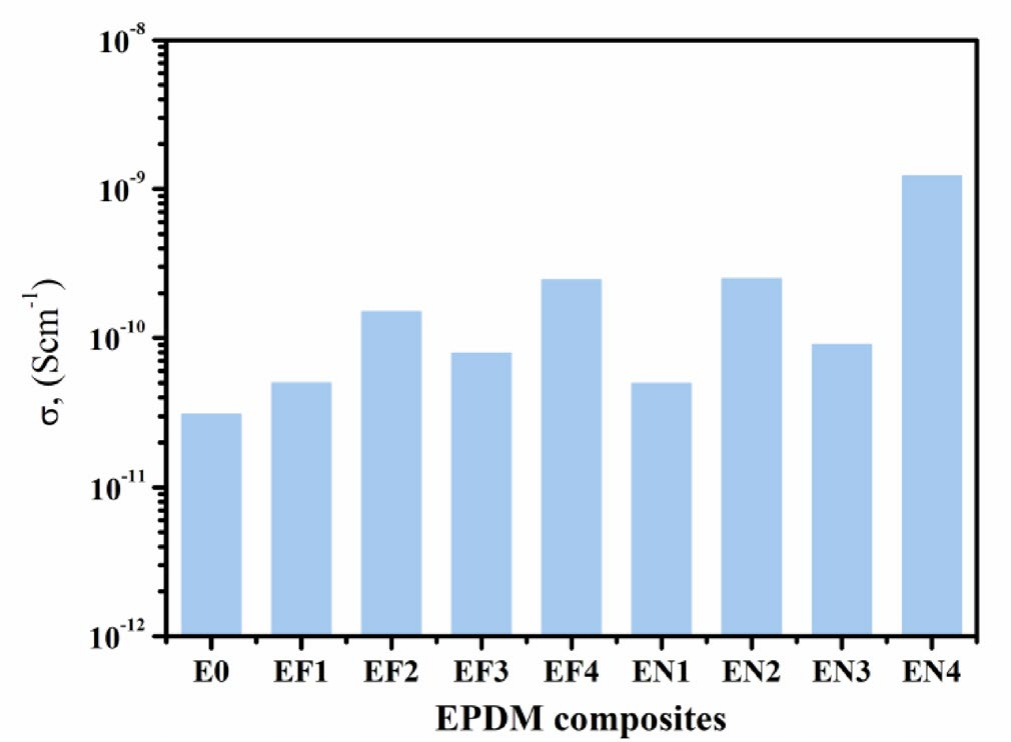
The variation of DC-conductivity (σdc) at 30 °C for EPDM composites loaded with green activator system Fe(LSF) and Ni(LSF) hybrids.

In addition to the dielectric characterization, the direct current DC-conductivity (σdc) of EPDM composites was assessed, providing insights into the static charge transport mechanisms influenced by green activators shape and dispersion. Figure 12 illustrates the fluctuation in DC-conductivity (σdc) throughout a range of EPDM-based composites; the electrical conductivity profiles of EPDM composites reveal that the system of activator and the changes in the microstructure have a big effect on them. EF_**4**_ and EN_**4**_ are the Fe(LSF)- and Ni(LSF)-based hybrids with the highest conductivity values, respectively. EF_**4**_ doesn’t have any ZnO or stearic acid in it, and it has a lot of Fe(LSF) hybrid, which probably makes the ingredients spread out unevenly and makes the interfacial areas more difficult.

These modest modifications in the structure make it easier for small polarons to migrate between Fe^2^⁺ and Fe^3^⁺ sites, which make localized charge transfer, better^71^. In the EN4 formulation, the elevated loading of Ni(LSF) hybrid (7 phr) significantly enhances the formation of multiphase interfaces within the EPDM matrix. These interfaces arise from the heterogeneous distribution of organic (lignin/fatty acid) and inorganic (NiO/silica) domains, which differ in conductivity and permittivity. This heterogeneity facilitates Maxwell–Wagner–Sillars (MWS) interfacial polarization**,** wherein charge carriers accumulate at the interfaces under an alternating electric field, leading to enhanced dielectric response at low frequencies. Simultaneously, the increased filler concentration promotes the formation of continuous or near-continuous conductive pathways, thereby lowering the percolation threshold and enabling percolative conduction**.** This dual mechanism-interfacial polarization and percolation-synergistically contributes to the higher electrical conductivity observed in EN_**4**_, as evidenced by its higher permittivity and conductivity values across the measured frequency range^72^. The EPDM composites obtained in this study presented dc-electrical conductivities of 10^**–12**^ to 10^**–9**^ S/cm. Thus, they effectively function as dielectrics in advanced dielectric systems. The conductivity profile, combined with significant interfacial polarization and tailored activator-matrix interactions, enables their application in high-voltage insulation systems^73^. They may be also suitable for flexible substrates in printed electronics in addition to antistatic applications^74,75^. However, the obtained results underscore the need of optimizing composition and altering microstructure to develop EPDM-based materials capable of multifunctionality for emerging technologies requiring regulated electrical insulation and robust dielectric performance.

This study introduces bio-derived hybrid activators (Fe(LSF) and Ni(LSF)) hybrids synthesized from rice straw black liquor as sustainable substitutes for conventional ZnO-stearic acid in EPDM vulcanization. The work uniquely correlates the hybrids’ microstructure with their dual functionality Fe(LSF) enhancing mechanical strength and Ni(LSF) improving dielectric performance offering a green, waste-to-value approach for multifunctional rubber composites.

## Conclusion

For this study, based bio hybrid additives, sourced from the black liquor by-product of rice straw pulping, was used as a substitute of conventional additives ZnO and stearic acid in the standard EPDM formulations. FTIR and XRF characterization validated the successful fabrication of Fe(LSF) and Ni(LSF) hybrids, revealing that Fe(LSF) contains greater amounts of lignin and silica than Ni(LSF). TEM and SEM revealed nano-scale morphology with particles overlapping in both hybrids. EDAX mapping also exhibited almost uniform elemental dispersion. Vulcanization tests showed that the hybrid accelerated the curing rate of the composites. The mechanical studies revealed that the tensile strength of 5 phr Fe(LSF) and Ni(LSF) hybrids caused an increase in their tensile strength to 5.57 MPa and 2.75 MPa, respectively, as compared to the control sample. The dielectric behavior of EPDM composites with Ni(LSF) and Fe(LSF) hybrids as green activators is influenced by hybrid composition, charge movement, and interface interactions. Meanwhile, the permittivity and dielectric loss of Ni(LSF) hybrid are enhanced by the inclusions of Ni^2^⁺ phases, vacancy-driven hopping, as well as interfacial polarization. This suggests that Fe(LSF) reinforces mechanical properties, whereas Ni(LSF) provides superior dielectric characteristics and potential aging stability. The dielectric performance of EPDM composites can be effectively enhanced by controlling microstructural factors such as green activators distribution, oxide composition, and matrix interaction. These engineered EPDM composites may have potential applications in insulation, antistatic devices, and flexible electronics. Ni(LSF) hybrid, performed better, suggesting a sustainable alternative activator for conductive rubber formulations.

## Data Availability

All data is provided within the manuscript.

## Abbreviations

(LSF): (Lignin/ silica/fatty acid)
Fe(LSF): Ferric(lignin/silica/fatty acid)
Ni(LSF): Nickel(lignin/silica/fatty acid)
EPDM: Ethylene propylene diene monomer
XRF: X-ray fluorescence spectroscopy
XRD: X-ray diffraction
SEM: Scanning electron microscopy
TEM: Transmission electron microscopy
(FTIR): Fourier transform infrared spectra
ZnO: Zinc oxide
CDs: Carbon dots
SBR: Styrene-butadiene rubber
TMQ: 2, 2, 4-Trimethyl-1, 2-dihydroquinoline
IPPD: N-isopropyl-N′-phenyl-P-phenylene diamine
NBR: Nitrile butadiene rubber
(Cu-LSF): Cupper(lignin/silica/fatty acid
(Zn–LSF): Zinc(lignin/silica/fatty acid)
Al(LSF): Aluminum(lignin/silica/fatty acid
TS: Tensile strength
EB: Elongation at break
CRI: Cure rate index
t_s2_: Scorch time
t_c50_: The time of cure at torque 50%
tc_90_: Optimum cure time
AG: Aging coefficient
